# Developmental stability of task-rest neural efficiency in youth using a threat and cognitive control task

**DOI:** 10.3389/fnhum.2026.1839961

**Published:** 2026-07-06

**Authors:** Parmis Khosravi, Julia O. Linke, Anjali D. Poe, Chase Antonacci, Reut Naim, Elise Cardinale, Katharina Kircanski, Anderson Winkler, Nathan Fox, Daniel S. Pine, Simone P. Haller

**Affiliations:** 1 Emotion and Development Branch, National Institute of Mental Health, National Institutes of Health, Bethesda, MD, United States; 2 Department of Psychology, Johannes-Gutenberg University, Mainz, Germany; 3 Leibniz Institute for Resilience Research, Mainz, Germany; 4 Department of Psychology, Stony Brook University, Stony Brook, NY, United States; 5 Department of Psychology, Stanford University, Stanford, CA, United States; 6 School of Psychological Sciences, Tel-Aviv University, Tel Aviv-Yafo, Israel; 7 Sagol School of Neuroscience, Tel-Aviv University, Tel Aviv-Yafo, Israel; 8 Department of Psychology, Catholic University of America, Washington, DC, United States; 9 Department of Psychology, University of Southern California, Los Angeles, CA, United States; 10 Division of Human Genetics, School of Medicine, University of Texas Rio Grande Valley, Brownsville, TX, United States; 11 Department of Human Development and Quantitative Methodology, University of Maryland, College Park, MD, United States

**Keywords:** children and adolescents, cognitive control, fMRI, neural efficiency, resting state, threat processing

## Abstract

Behaviors arise from coordinated neural activity across diverse spatial and temporal scales. Prior work has linked better task performance and cognitive functioning to patterns of global network connectivity requiring minimal reconfiguration when switching between task demands. This metric indexing similarity in functional connectivity across task and rest has been termed “neural efficiency.” Here we assess stability of neural efficiency over approximately 3 years in adolescence, specificity across two task-rest combinations and associations with anxiety. At approximately age 16 and/or 19, 95 participants completed a resting state scan alongside a cognitive control and/or threat task. Neural efficiency was quantified as partial correlations between intrinsic and task-related functional connectivity patterns across the whole brain. We tested temporal stability across the three-year interval, as well as associations with task performance and anxiety across the two task-rest combinations at the two time points. Neural efficiency values remained relatively stable from mid to late adolescence (ICC[3,1] = 0.51–0.58). The cognitive control task showed higher values than the threat task. Across tasks, neural efficiency was associated with better performance (i.e., reduced interference), although not consistently (*r* = −0.19, *p* = 0.26 – *r* = −0.37, *p =* 0.021). These effects did not survive correction for multiple testing. No associations were found between neural efficiency and self/parent-reported anxiety. In sum, the metric shows moderate developmental stability and associations with task performance. Task features impact neural efficiency. Given small sample sizes, findings need to be interpreted cautiously.

## 1 Introduction

Recent fMRI studies combined task-based and resting-state data to extract a metric termed *neural efficiency*, computed as pairwise correlations between vectorized connectivity matrices. Neural efficiency relates to behavior, cognition, and symptoms in pediatric and adult samples (Harrewijn et al., 2020; Lee et al., 2025; Linke et al., 2025; Schultz and Cole, 2016). Here we extend prior work by assessing, (i) stability across adolescence and (ii) specificity across two tasks-rest combinations.

Functional connectivity during rest and task is highly correlated. Subtle changes in connectivity support task demands, with smaller reconfigurations associated with higher intelligence and better task performance (Schultz and Cole, 2016). Accordingly, such minimal reconfiguration has been interpreted as reflecting a functional architecture that is preconfigured to meet diverse cognitive demands (Thiele et al., 2022). However, this literature remains limited, and alternative interpretations are possible (Ai et al., 2026; Lee et al., 2025; Linke, 2026). One important unanswered question concerns the trait-like nature of the measure, particularly across adolescence. A prior study established moderate-to-good temporal stability across 8–12 weeks in healthy adolescents aged ~12.5 years (ICC = 0.65). However, with marked maturational changes in long- and short-range connections in association cortical areas (Park et al., 2022), it is unclear whether this metric remains stable across adolescent development.

Another related set of questions concerns whether task features influence overall levels of efficiency, their stability, as well as associations with task performance and individual differences. Efficiency in functional connectivity may differ between tasks with neutral stimuli that place demands on cognitive control and flexibility, as compared to tasks predominantly with emotionally valenced stimuli involving fewer task demands. Thus, it remains unclear if efficiency in adolescence differs across tasks differing in design features. Prior work suggests relevance of this metric to mood disorder symptoms; however, results have been mixed, with some studies finding positive associations between neural efficiency and internalizing symptoms, and other studies finding the reverse or no associations (Harrewijn et al., 2020; Lee et al., 2025; Linke et al., 2025). Discrepancies are likely due to methodological and sample differences; additional work is sorely needed to assess robustness of associations. Here, we use longitudinal data collected from mid and late adolescents to evaluate these two sets of questions.

## 2 Methods

### 2.1 Participants

Participants were recruited from a longitudinal study on temperamental risk for anxiety (Hane et al., 2008). At ~16 and ~19 years participants completed a resting state scan alongside a threat task (emotional Dot-probe) and/or cognitive control (Flanker) task. A total of 95 participants contributed data across the two tasks and sessions, resulting in a total of 162 scanning sessions. Eighty-six (86) youth completed at least one Flanker/rest session, 76 youth completed at least one emotional Dot-probe/rest session. Twenty-one (21) participants completed Flanker/rest at both time points, 20 youth completed the Dot-probe/rest at both time points. Written consent/assent from parents/children was obtained prior to the initiation of any study procedures; families received monetary compensation for their participation. The National Institute of Mental Health (NIMH) and University of Maryland Institutional Review Boards approved the study. For sample characteristics see Table 1. For details on participant exclusion see Supplementary materials.

**Table 1:** Sample characteristics.

Variable	16-year		19-year	
	Flanker	Dot-probe	Flanker	Dot-probe
*n*	43	36	43	40
Sex *N*%				
Female	22 (51%)	20 (56%)	17 (40%)	20 (50%)
Male	21 (49%)	16 (44%)	26 (61%)	20 (50%)
Race *N*%				
Black/African American	12 (28%)	8 (22%)	8 (19%)	9 (23%)
Asian American	1 (2%)	–	–	–
White	27 (63%)	25 (69%)	29 (67%)	25 (63%)
Multiple races	3 (7%)	3 (8%)	5 (12%)	5 (13%)
Unkown	–	–	1 (2%)	1 (3%)
Ethnicity *N*%				
Latino/hispanic	4 (9%)	4 (11%)	4 (9%)	4 (1%)
Not Latino/hispanic	39 (91%)	32 (89%)	39 (91%)	36 (90%)
Mother’s education *N*%				
High school graduate	7 (16%)	8 (22%)	6 (14%)	7 (18%)
College graduate	9 (21.%)	9 (25%)	12 (28%)	12 (30%)
Graduate school graduate	12 (28%)	11 (31%)	12 (28%)	12 (30%)
Age *M* (SD)	16.31 (0.69)	16.37 (0.71)	19.51 (0.95)	19.23 (0.73)
Anxiety score *M* (SD)				
SCARED child-rated	14.20 (13.57)	14.67 (14.46)	12.00 (10.59)	10.90 (9.36)
SCARED parent-rated	10.07 (9.06)	10.42 (9.31)	–	–
Task data *M* (SD)				
Reaction time	415.15 (46.14)	496.70 (63.65)	401.37 (47.86)	466.55 (49.11)
Accuracy	0.87 (0.05)	0.92 (0.04)	0.88 (0.06)	0.93 (0.06)

SCARED, Screen for Anxiety Related Disorders.

Mother’s education data missing for *n* = 15 participants at the 16-year time point, *n* = 13 participants at the 19-year time point (Flanker data only), *n* = 9 participants at the 19-year timepoint (Dot-probe data only).

SCARED data missing for *n* = 2 participants at the 16-year timepoint (child- and parent-rated, Dot-probe data only); *n* = 16 participants at 19-year timepoint (child-rated Flanker data only); *n* = 20 participants at the 19-year timepoint (child-rated Dot-probe data only).

### 2.2 fMRI acquisition and task

All data were acquired on 3 T scanners. Each participant completed an 8-min multi-echo resting state scan and a structural scan for co-registration with the functional data. Participants also completed an Eriksen Flanker task and/or an emotional Dot-probe task. In the Flanker task, participants were asked to respond to a central arrow flanked by arrows pointing either in the same (congruent) or opposite direction (incongruent); the Dot-probe task required participants to indicate the direction of an arrow replacing threatening, happy or neutral face pairs. For acquisition, task and procedural details, see Supplementary materials.

### 2.3 fMRI data preprocessing

Procedures followed Linke et al. (2025). fMRI data were preprocessed using fMRIPrep version 23.1.4 software (Esteban et al., 2019a,b). To retain a sizable sample, we applied an exclusion threshold of <70% of frames with framewise displacement of <0.5 mm for both task and rest data. The time course of 100 cortical (Schaefer et al., 2018) and 16 subcortical regions was extracted for task and rest data; head motion, white matter and cerebrospinal fluid. For task data, task events (Flanker arrows and Dot-probe face pairs and probes) were additionally regressed out. As partial correlation coefficients have demonstrated higher test–retest reliability than full correlations in prior work (Linke et al., 2025), two first-level 116×116 functional connectivity matrices represented partial correlations measuring direct functional connectivity between pairs of regions for task and rest scans per participant. Neural efficiency was calculated as a Pearson correlation of the vectorized upper diagonal elements of the task and rest functional connectivity matrices. For additional preprocessing details, correlations between similarity values computed with and without additionally regressing out global signal and correlations between the two task-rest combinations, see Supplementary materials.

### 2.4 Group-level models

#### 2.4.1 Developmental stability

We used intra-class coefficients with a consistency formulation (ICC[3,1]) to assess stability of neural efficiency across the two time points (age ~16 and ~19) for each task-rest combination.

#### 2.4.2 Differences between tasks and association with task performance

We used a paired samples *t*-test to assess differences in neural efficiency values between the task/rest combinations at each time point. We then examined associations between neural efficiency and the incongruent-congruent reaction time difference value (Flanker) and incongruent-congruent angry reaction time value (i.e., a threat bias index from the emotional Dot-probe) using Pearson correlations.

#### 2.4.3 Association with anxiety

We examined associations between neural efficiency and anxiety as measured by the child- and parent-report versions of the Screen for Child Anxiety Related Disorder (SCARED) (Birmaher et al., 1997). At the 16-year time point, both child and parent ratings were available, whereas at the 19-year time point only child-reported data were collected.

To correct for multiple comparisons within each task, *p*-values were adjusted using the Benjamini-Hochberg procedure (False Discovery Rate, FDR) for five designs per task (two behavioral correlations [i.e., 16- and 19-year time point] and three correlations with anxiety scores [i.e., parent- and child-rated anxiety at age- and child-rated anxiety at age 19-year]).

## 3 Results

### 3.1 Developmental stability

**Figure 1:**
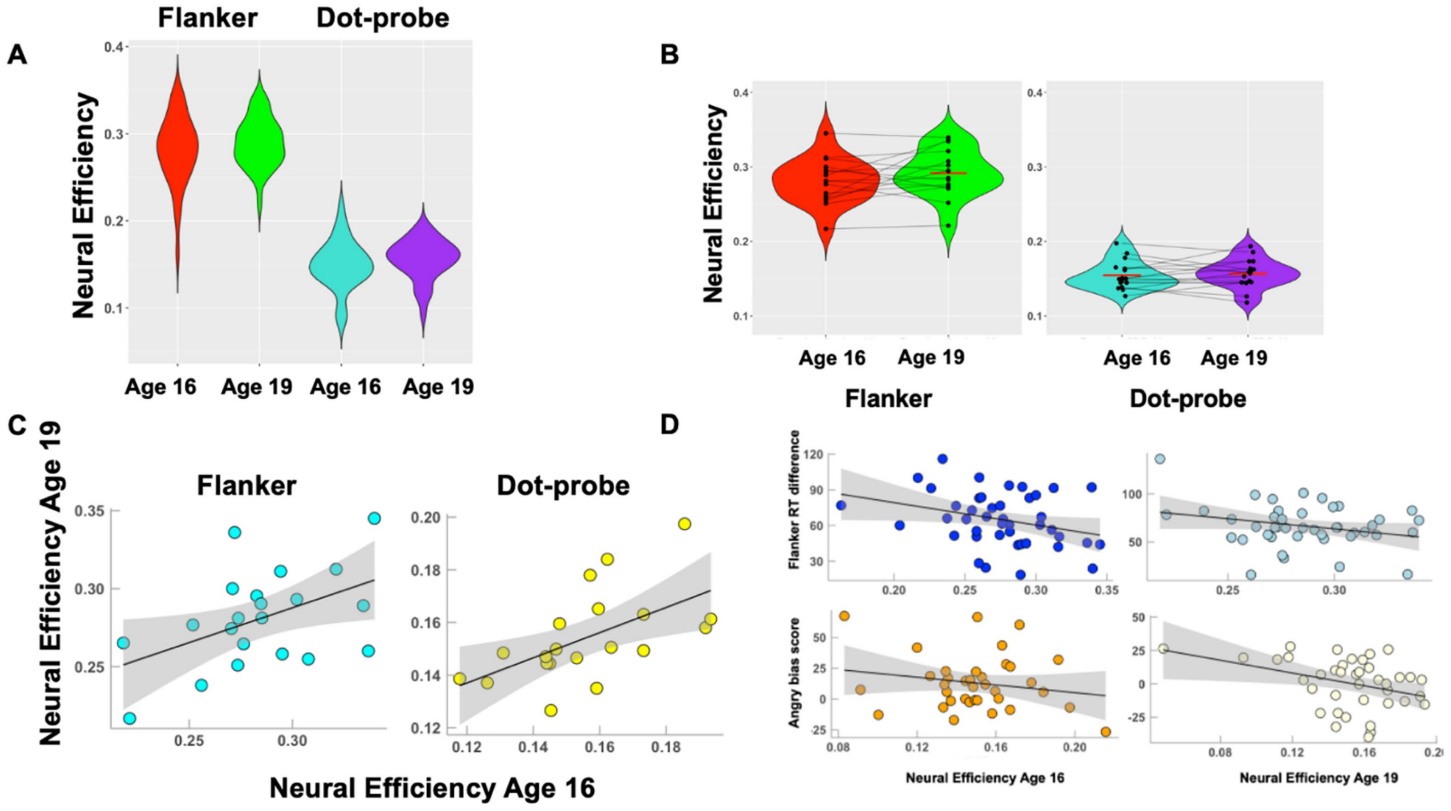
**(A)** At both time points the Flanker task showed higher neural efficiency values than the emotional Dot-probe task. **(B)** Neural efficiency was relatively stable from mid to late adolescence. **(C)** Correlations between neural efficiency values across time points for each task. **(D)** Associations between task behavior and neural efficiency.

Neural efficiency showed moderate stability from mid to late adolescence (i.e., from ~16-19-years) in both the Flanker (ICC = 0.51, 95% CI [0.20, 0.77]) and emotional Dot-probe task (ICC = 0.58, 95% CI [0.30, 0.81]; see Figures 1B,C).

### 3.2 Differences between tasks and association with task performance

At both time points, the Flanker task showed higher neural efficiency values than the emotional Dot-probe task (age 16: *t*(32) = 25.97, *p* < 0.001; age 19: *t*(33) = 25.81, *p* < 0.001, see Figure 1A). Thus, greater-rest-task differences occurred for the task containing negative-valence stimuli. Neural efficiency negatively correlated with Flanker reaction time difference, at age 16-years (*r*(41) = −0.31, *p* = 0.042, *p*_FDR_ = 0.20) with a similar trend at 19-years (*r*(41) = −0.27, *p* = 0.0797, *p*_FDR_ = 0.20). Unlike prior work with the sample at an earlier time point (Harrewijn et al., 2020), for the emotional Dot-probe task, we found no significant association between neural efficiency and threat bias at the 16-year time point (*r*(34) = −0.19, *p* = 0.268, *p*_FDR_ = 0.669), though the association was significant at age 19 (*r*(38) = −0.365, *p* = 0.021, *p*_FDR_ = 0.103, see Figure 1D).

### 3.3 Associations with anxiety

No significant associations emerged for either task with anxiety scores at either age (Flanker 16-year time point: *r*_child_(41) = 0.183, *p* = 0.24, *p*_FDR_ = 0.398, *r*_parent_(41) = 0.09, *p* = 0.58, *p*_FDR_ = 0.579; 19-year time point: *r*_child_(27) = −0.13, *p* = 0.52, *p*_FDR_ = 0.579; emotional Dot-probe 16-year time point: *r*_child_ (32) = 0.095, *p* = 0.59, *p*_FDR_=. 967, *r*_parent_(32) = 0.01, *p* = 0.97, *p*_FDR_ = 0.967; 19-year time point: *r*_child_ (27) = 0.01, *p* = 0.95, *p*_FDR_ = 0.967).

## 4 Discussion

Three key findings emerged from the present work. First, analyses for the cognitive control task generated higher neural efficiency values than the emotional Dot-probe task, though both tasks generated relatively stable estimates of efficiency from mid to late adolescence. Second, task behavior related to neural efficiency for both tasks with modest consistency. Third, no association emerged between neural efficiency and self/parent-reported anxiety.

Our results extend previous work on task-invariant signal in distributed brain networks (Cole et al., 2014; Geerligs et al., 2015; Krienen et al., 2014). The significantly higher configurational similarity between the Flanker/rest combination compared to the emotional Dot-probe/rest combination may suggest that stimulus-driven affective and salience processes deviate more from intrinsic functional organization than processes engaging cognitive control networks that may already be active during rest. However, because these tasks differ in both stimulus features and task demands, we cannot determine which aspects account for the observed differences in similarity values. Future studies with larger samples will be needed to disentangle the contributions of these and other task features (i.e., length of task events and inter-trial rest intervals) on estimates of neural efficiency.

ICC values across the ~3 year time interval resembled those reported for much shorter time spans (Linke et al., 2025). Prior cross-sectional work has found a small effect of age on neural efficiency in the reward network across a larger age range (9 to 20)—a relationship that was attenuated by early life stress (Lee et al., 2025). As the brain reorganizes from childhood through adolescence—with networks becoming more segregated and integrated as long-range connections strengthen and cognitive control systems mature (Fair et al., 2007; Hagmann et al., 2010; Marek et al., 2015; Park et al., 2022)—we would expect corresponding increases in the stability and efficiency in network organization, and consequently shifts in neural efficiency. However, many of these developmental changes may emerge prior to our earliest sampling time point. Pre-adolescent children, who exhibit more variable connectivity patterns (Marek et al., 2015; Sanders et al., 2023), may require greater network reconfiguration to support task performance. Characterizing developmental changes in neural efficiency, as well as their task and network specificity, therefore remains an important direction for future research.

Prior work relates higher neural efficiency to task performance (Harrewijn et al., 2020; Linke et al., 2025; Schultz and Cole, 2016). In our study, a smaller RT difference between incongruent and congruent trials for both the Flanker and Dot-probe task (i.e., a smaller interference effect) related to higher neural efficiency values. While some inconsistency manifested in statistical significance across ages, similar directionality and magnitudes emerged across cognitive performance-neural efficiency associations. Effects did not survive corrections for multiple testing; replications are needed in larger samples to evaluate the robustness of performance-efficiency associations across tasks and age groups. Similarity is a coarse, global metric, collapsing many connection-level changes into a single value – only a subset of connection changes may be behaviorally relevant given a particular task context (Petrican and Levine, 2018). Of note, previous analyses in the 12-year cohort found different patterns between task performance and neural efficiency for the Dot-probe task. Inconsistency with current patterns could relate to the poor reliability of measures (Xu et al., 2025).

Null results with regards to associations between neural efficiency and anxiety scores are consistent with prior work in this sample at the 12 year time point (Harrewijn et al., 2020). Linke and colleagues (Linke et al., 2025) found a small but significant negative association between parent-rated anxiety scores and neural efficiency in a sample of youth with and without anxiety disorders. It is plausible that individual differences shape this association, such that for some individuals higher similarity reflects efficiency, while for others—particularly in task requiring flexibility—greater reconfiguration may be advantageous, especially in adolescence when network reorganization is ongoing. Detecting such effects, or disentangling potential heterogeneity in this relationship, will require larger samples with a broader range of anxiety scores.

In sum, we found novel evidence for task feature dependency of neural efficiency markers, moderate developmental stability and associations with task performance. However, given the small sample size, the findings need to be interpreted cautiously.

## Data Availability

In accordance with the NIH Data Management and Sharing Policy, individual imaging data collected at the NIH will be shared in a public repository for participants who consented to public data sharing. These data will be available immediately following publication through OpenNeuro: https://openneuro.org/datasets/ds007812. Some individual differences data were acquired at the University of Maryland. These data, with matching identifiers, are available upon reasonable request from SH or NF, respectively, but are not publicly available due to privacy restrictions. Analytical code is available on GitHub: https://github.com/NIMH-SDAN/Neural.Efficiency.2025.

## Author disclaimer

The contributions of the NIH author(s) are considered Works of the United States Government. The findings and conclusions presented in this paper are those of the author(s) and do not necessarily reflect the views of the NIH or the U.S. Department of Health and Human Services.

## References

[ref1] Ai Y. Xie J. Hou X. (2026). Reconsidering the interpretive basis of neural efficiency as a biomarker in Pediatric anxiety. Am. J. Psychiatry 183, 273–273. doi: 10.1176/appi.ajp.20251115, 41917707

[ref2] Birmaher B. Khetarpal S. Brent D. Cully M. Balach L. Kaufman J. . (1997). The screen for child anxiety related emotional disorders (SCARED): scale construction and psychometric characteristics. J. Am. Acad. Child Adolesc. Psychiatry 36, 545–553. doi: 10.1097/00004583-199704000-00018, 9100430

[ref3] Cole M. W. Bassett D. S. Power J. D. Braver T. S. Petersen S. E. (2014). Intrinsic and task-evoked network architectures of the human brain. Neuron 83, 238–251. doi: 10.1016/j.neuron.2014.05.014, 24991964 PMC4082806

[ref4] Esteban O. Blair R. W. Nielson D. M. Varada J. C. Marrett S. Thomas A. G. . (2019a). Crowdsourced MRI quality metrics and expert quality annotations for training of humans and machines. Sci. Data 6:30. doi: 10.1038/s41597-019-0035-4, 30975998 PMC6472378

[ref5] Esteban O. Markiewicz C. J. Blair R. W. Moodie C. A. Isik A. I. Erramuzpe A. . (2019b). fMRIPrep: a robust preprocessing pipeline for functional MRI. Nat. Methods 16, 111–116. doi: 10.1038/s41592-018-0235-4, 30532080 PMC6319393

[ref6] Fair D. A. Dosenbach N. U. Church J. A. Cohen A. L. Brahmbhatt S. Miezin F. M. . (2007). Development of distinct control networks through segregation and integration. Proc. Natl. Acad. Sci. 104, 13507–13512. doi: 10.1073/pnas.0705843104, 17679691 PMC1940033

[ref7] Geerligs L. Rubinov M. Henson R. N. (2015). State and trait components of functional connectivity: individual differences vary with mental state. J. Neurosci. 35, 13949–13961. doi: 10.1523/JNEUROSCI.1324-15.2015, 26468196 PMC4604231

[ref8] Hagmann P. Sporns O. Madan N. Cammoun L. Pienaar R. Wedeen V. J. . (2010). White matter maturation reshapes structural connectivity in the late developing human brain. Proc. Natl. Acad. Sci. 107, 19067–19072. doi: 10.1073/pnas.1009073107, 20956328 PMC2973853

[ref9] Hane A. A. Fox N. A. Henderson H. A. Marshall P. J. (2008). Behavioral reactivity and approach-withdrawal bias in infancy. Dev. Psychol. 44, 1491–1496. doi: 10.1037/a0012855, 18793079 PMC2575804

[ref10] Harrewijn A. Abend R. Linke J. Brotman M. A. Fox N. A. Leibenluft E. . (2020). Combining fMRI during resting state and an attention bias task in children. NeuroImage 205:116301. doi: 10.1016/j.neuroimage.2019.116301, 31639510 PMC6911838

[ref11] Krienen F. M. Yeo B. Buckner R. L. (2014). Reconfigurable task-dependent functional coupling modes cluster around a core functional architecture. Philos. Trans. R. Soc. B Biol. Sci. 369:20130526. doi: 10.1098/rstb.2013.0526, 25180304 PMC4150301

[ref12] Lee Y. Yuan J. P. Winkler A. M. Kircanski K. Pine D. S. Gotlib I. H. (2025). Task–rest reconfiguration efficiency of the reward network across adolescence and its association with early life stress and depressive symptoms. J. Am. Acad. Child Adolesc. Psychiatry 64, 290–300. doi: 10.1016/j.jaac.2024.04.018, 38878818 PMC11638404

[ref13] Linke J. O. (2026). Reconsidering the interpretive basis of neural efficiency as a biomarker in Pediatric anxiety: response to Ai and colleagues. Am. J. Psychiatry 183, 274–274. doi: 10.1176/appi.ajp.20260131, 41917708

[ref14] Linke J. O. Naim R. Haller S. P. Khosravi P. Scheinberg B. Byrne M. E. . (2025). Reduced threat-related neural efficiency: a possible biomarker for pediatric anxiety disorders. Am. J. Psychiatry 183, 48–57. doi: 10.1176/appi.ajp.20241043 41058235 PMC12614269

[ref15] Marek S. Hwang K. Foran W. Hallquist M. N. Luna B. (2015). The contribution of network organization and integration to the development of cognitive control. PLoS Biol. 13:e1002328. doi: 10.1371/journal.pbio.1002328, 26713863 PMC4694653

[ref16] Park B.-Y. Paquola C. Bethlehem R. A. Benkarim O. Neuroscience in Psychiatry Network (NSPN) Consortium Mišić B. . (2022). Adolescent development of multiscale structural wiring and functional interactions in the human connectome. Proc. Natl. Acad. Sci. 119:e2116673119. doi: 10.1073/pnas.2116673119, 35776541 PMC9271154

[ref17] Petrican R. Levine B. T. (2018). Similarity in functional brain architecture between rest and specific task modes: a model of genetic and environmental contributions to episodic memory. NeuroImage 179, 489–504. doi: 10.1016/j.neuroimage.2018.06.057, 29936311

[ref18] Sanders A. F. Harms M. P. Kandala S. Marek S. Somerville L. H. Bookheimer S. Y. . (2023). Age-related differences in resting-state functional connectivity from childhood to adolescence. Cereb. Cortex 33, 6928–6942. doi: 10.1093/cercor/bhad011, 36724055 PMC10233258

[ref19] Schaefer A. Kong R. Gordon E. M. Laumann T. O. Zuo X.-N. Holmes A. J. . (2018). Local-global parcellation of the human cerebral cortex from intrinsic functional connectivity MRI. Cereb. Cortex 28, 3095–3114. doi: 10.1093/cercor/bhx179, 28981612 PMC6095216

[ref20] Schultz D. H. Cole M. W. (2016). Higher intelligence is associated with less task-related brain network reconfiguration. J. Neurosci. 36, 8551–8561. doi: 10.1523/JNEUROSCI.0358-16.2016, 27535904 PMC4987432

[ref21] Thiele J. A. Faskowitz J. Sporns O. Hilger K. (2022). Multitask brain network reconfiguration is inversely associated with human intelligence. Cereb. Cortex 32, 4172–4182. doi: 10.1093/cercor/bhab473, 35136956 PMC9528794

[ref22] Xu I. Passell E. Strong R. W. Grinspoon E. Jung L. Wilmer J. B. . (2025). No evidence of reliability across 36 variations of the emotional dot-probe task in 9,600 participants. Clin. Psychol. Sci. 13, 261–277. doi: 10.1177/21677026241253826, 40151297 PMC11949442

