# Supplementary material for: Developmental stability of task-rest neural efficiency in youth using a threat and cognitive control task
Source: Front Hum Neurosci. 2026 Jul 6;20:1839961. doi: 10.3389/fnhum.2026.1839961 (PMC13381436; doi:10.3389/fnhum.2026.1839961)
Supplement: Supplementary file 1 [file Data_Sheet_1.DOCX]

Supplemental materials

**Developmental stability of task-rest neural efficiency in youth using a threat and cognitive control task**

1. **Participant exclusions**

Participants were excluded for not meeting thresholds for behavioral performance (>70% accuracy) or excessive motion (<70% frames with framewise displacement of <0.5mm for both task and rest). At the 16-year time point for the Flanker/rest combination, one person was excluded for poor accuracy on the task, none for excessive motion. At the 19-year time point, one person was removed for poor accuracy, and three were excluded for excessive motion. For the emotional Dot-probe, no participant was removed for poor accuracy, but four participants were excluded for excessive motion. One participant was excluded for poor accuracy and four participants for excessive motion at the 19-year time point.

| **Criterion** | **Flanker** | | **Dot-probe** | |
| --- | --- | --- | --- | --- |
|  | 16-year | 19-year | 16-year | 19-year |
| Accuracy | 1 | 1 | 0 | 1 |
| Motion | 0 | 3 | 4 | 4 |
| Total exclusions | 1 | 4 | 4 | 5 |
| Total sample size before exclusion | 44 | 47 | 40 | 45 |
| Total sample size after exclusion | 43 | 43 | 36 | 40 |

1. **fMRI tasks and rest specifications**

**Rest scan**

Participants underwent a 490s (~8 min) resting state fMRI scan. A white fixation cross was displayed on a black background. Participants were asked to keep their eyes open.

**Flanker task**

On each trial, participants are presented with a jittered fixation cross followed by a display of five arrows. The center arrow is flanked by arrows either pointing in the same (congruent) or opposite (incongruent) direction. The task was composed of 90 congruent and 90 incongruent trials (45 per direction), presented at random across four task runs. Participants received feedback on their performance after the completion of each block (three blocks per task run); “be more accurate” for accuracy under 75%, “respond faster” for accuracy between 75% and 90%, and “good job” for accuracy above 90%. The task also included 45 “null” trials (1900 ms each, a trial consisting only of a blank screen) to increase jitter between trials. See Figure S1A for a task schematic.

**Emotional Dot-probe task**

On each trial, following a fixation cross, a pair of faces was presented horizontally. Face pairs were either happy-neutral, angry-neutral or neutral-neutral. An arrow probe subsequently replaced one of the two faces, either on the same side as the emotional face (congruent trial) or the opposite (neutral) side (incongruent trial). Emotions and probe locations were counterbalanced across emotion pairs. Images of faces were taken from the NimStim set [1]. Participants were instructed to indicate the direction of the arrow (up or down) via button press. The task was composed of 48 trials of each emotion-probe combination (i.e., 48 angry congruent, 48 angry incongruent, 48 happy congruent, 48 happy incongruent trials) and 96 neutral-neutral trials. The task also included 48 null trials. Participants completed 2 runs of the task. See Figure S1B for a task schematic.


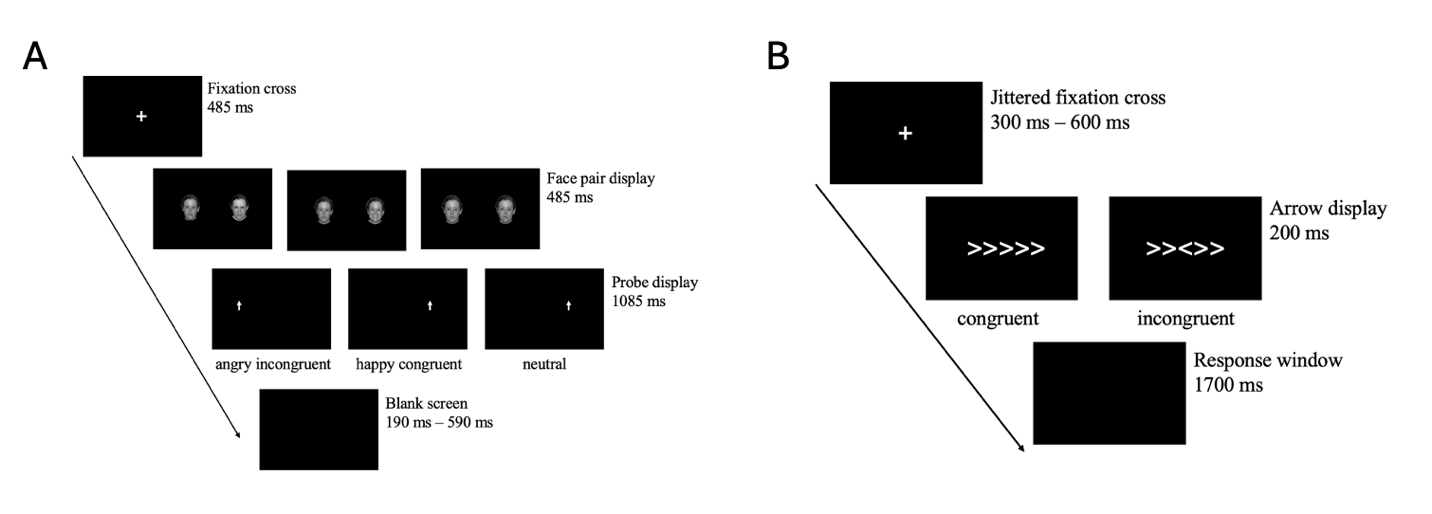


Figure 1S. A Emotional Dot-probe task schematic B. Flanker task schematic

1. **Acquisition parameters**

Neuroimaging data were acquired on two identical 3T General Electric SIGNA Scanners (GE Medical Systems Milwaukee, WI) using a 32-channel head coil. After a localizer scan, an automated shim procedure was used to calibrate the magnetic field and reduce signal drop out from susceptibility artifact.

**Rest scan**

One functional run consisting of 196 time points each were acquired using 32 contiguous, interleaved axial slices at 3 mm each covering the whole brain. Scans used a multi-echo planar imaging sequence with the following specifications: repetition time (TR) =2500 ms, Echo Time (TE) 1/2/3/4=15.2/28.8/42.4/56.0 s, flip angle 80°, field of view 24, matrix size 64x64, resulting in 3.75x3.75x4mm voxels.

A sagittal, high resolution, magnetization-prepared rapid acquisition gradient echo (MPRAGE) sequence scan was collected for co-registration with the functional data. This structural scan was acquired with the following specifications: TI/TE = 900/min full, 1-mm slices, flip angle = 7°, 25.6 field of view, 256 × 256 matrix. The same structural scan was used to co-register the functional task runs (i.e., Flanker and emotional Dot-probe task).

**Flanker task**

Four functional task runs consisting of 170 time points each were acquired using 42 contiguous, interleaved axial slices at 3mm each covered the whole brain. Scans used an echo-planer single shot gradient echo T2*-weighted with the following specifications: TR= 2000 ms, TE= 25, flip angle 60°, field of view 24, matrix size 96 × 96 resulting in 2.5x2.5x3 mm voxels. We discarded the first four volumes of each run to ensure that the scanner had reached longitudinal magnetization equilibrium.

**Emotional Dot-probe task**

Four functional task runs consisting of 111 time points each were acquired using 47 contiguous, interleaved axial slices at 3 mm each covered the whole brain. Scans used an echo-planar single shot gradient echo T2*-weighted with the following specifications: repetition time TR =2300 ms, TE = 25, flip angle 50°, field of view 24, matrix size 96 × 96, resulting in 2.5x.25x3 mm voxels. We discarded the first four images of each run to ensure that the scanner had reached longitudinal magnetization equilibrium.

1. **Additional procedural information**

Participants could complete the three scans in one to two visits. Both task scans were always completed in the same session. Task order was counterbalanced. For the Flanker task at age 16, participants completed task and rest scans between 1 and 134 days apart (*M*=20 days, *SD*=26 days); at age 19 all participants completed all three scans in the same session. For the Dot-probe task, participants completed task and rest scans between 0 and 90 days apart (*M*=15.3 days, *SD*=18.15 days); at age 18 all but three participants completed all scans in the same session (*M*=0.54 days, *SD*=2.42 days). Except for a single acquisition, each participant completed both task scans on the same scanner across both time points.

1. **Additional data analyses information**

**Task data cleaning**

For both tasks, trials were removed if they had reaction times <150 ms. Sessions were removed for poor accuracy (<70% correct responses).

**fMRI data processing**

This work utilized the computational resources of the NIH HPC Biowulf cluster (<http://hpc.nih.gov>). Motion quality thresholds were assessed using the fMRIPrep output. The following information was obtained from fMRIPrep html output:

Results included in this manuscript come from preprocessing performed using *fMRIPrep* 23.1.4 [2], which is based on *Nipype* 1.8.6 [3].

**Anatomical data preprocessing**

A total of 4 T1-weighted (T1w) images were found within the input BIDS dataset. All of them were corrected for intensity non-uniformity (INU) with N4BiasFieldCorrection [4], distributed with ANTs (version unknown) [5]. The T1w-reference was then skull-stripped with a *Nipype* implementation of the antsBrainExtraction.sh workflow (from ANTs), using OASIS30ANTs as target template. Brain tissue segmentation of cerebrospinal fluid (CSF), white-matter (WM) and gray-matter (GM) was performed on the brain-extracted T1w using fast (FSL 6.0.5) [6]. An anatomical T1w-reference map was computed after registration of 4 T1w images (after INU-correction) using mri_robust_template (FreeSurfer 7.3.2) [7]. Brain surfaces were reconstructed using recon-all (FreeSurfer 7.3.2) [8], and the brain mask estimated previously was refined with a custom variation of the method to reconcile ANTs-derived and FreeSurfer-derived segmentations of the cortical gray-matter of Mindboggle [9]. Volume-based spatial normalization to two standard spaces (MNI152NLin6Asym, MNI152NLin2009cAsym) was performed through nonlinear registration with antsRegistration (ANTs (version unknown)), using brain-extracted versions of both T1w reference and the T1w template. The following templates were selected for spatial normalization and accessed with *TemplateFlow* (23.0.0, [10]): *FSL’s MNI ICBM 152 non-linear 6th Generation Asymmetric Average Brain Stereotaxic Registration Model* [[11], TemplateFlow ID: MNI152NLin6Asym], *ICBM 152 Nonlinear Asymmetrical template version 2009c,* [12], [TemplateFlow ID: MNI152NLin2009cAsym].

**Rest**

For each of the rest BOLD runs found per subject across sessions, the following preprocessing was performed. First, a reference volume and its skull-stripped version were generated from the shortest echo of the BOLD run using a custom methodology of *fMRIPrep*. Head-motion parameters with respect to the BOLD reference (transformation matrices, and six corresponding rotation and translation parameters) are estimated before any spatiotemporal filtering using mcflirt (FSL , [13]). BOLD runs were slice-time corrected to 1.25s (0.5 of slice acquisition range 0s-2.5s) using 3dTshift from AFNI [14]. The BOLD time-series (including slice-timing correction when applied) were resampled onto their original, native space by applying the transforms to correct for head-motion. These resampled BOLD time-series will be referred to as *preprocessed BOLD in original space*, or just *preprocessed BOLD*. A T2^★^ map was estimated from the preprocessed EPI echoes, by voxel-wise fitting the maximal number of echoes with reliable signal in that voxel to a monoexponential signal decay model with nonlinear regression. The T2^★^/S_0_ estimates from a log-linear regression fit were used for initial values. The calculated T2^★^ map was then used to optimally combine preprocessed BOLD across echoes following the method described in [15]. The optimally combined time series was carried forward as the *preprocessed BOLD*. The BOLD reference was then co-registered to the T1w reference using bbregister (FreeSurfer) which implements boundary-based registration [16]. Co-registration was configured with six degrees of freedom. First, a reference volume and its skull-stripped version were generated using a custom methodology of *fMRIPrep*. Several confounding time-series were calculated based on the *preprocessed BOLD*: framewise displacement (FD), DVARS and three region-wise global signals. FD was computed using two formulations following Power (absolute sum of relative motions, [17]) and Jenkinson (relative root mean square displacement between affines, [13]). FD and DVARS are calculated for each functional run, both using their implementations in *Nipype* (following the definitions by [17]). The three global signals are extracted within the CSF, the WM, and the whole-brain masks. Additionally, a set of physiological regressors were extracted to allow for component-based noise correction (*CompCor*, [18]). Principal components are estimated after high-pass filtering the *preprocessed BOLD* time-series (using a discrete cosine filter with 128s cut-off) for the two *CompCor* variants: temporal (tCompCor) and anatomical (aCompCor). tCompCor components are then calculated from the top 2% variable voxels within the brain mask. For aCompCor, three probabilistic masks (CSF, WM and combined CSF+WM) are generated in anatomical space. The implementation differs from that of Behzadi et al. in that instead of eroding the masks by 2 pixels on BOLD space, a mask of pixels that likely contain a volume fraction of GM is subtracted from the aCompCor masks. This mask is obtained by dilating a GM mask extracted from the FreeSurfer’s *aseg* segmentation, and it ensures components are not extracted from voxels containing a minimal fraction of GM. Finally, these masks are resampled into BOLD space and binarized by thresholding at 0.99 (as in the original implementation). Components are also calculated separately within the WM and CSF masks. For each CompCor decomposition, the *k* components with the largest singular values are retained, such that the retained components’ time series are sufficient to explain 50 percent of variance across the nuisance mask (CSF, WM, combined, or temporal). The remaining components are dropped from consideration. The head-motion estimates calculated in the correction step were also placed within the corresponding confounds file. The confound time series derived from head motion estimates and global signals were expanded with the inclusion of temporal derivatives and quadratic terms for each [19]. Frames that exceeded a threshold of 0.5 mm FD or 1.5 standardized DVARS were annotated as motion outliers. Additional nuisance timeseries are calculated by means of principal components analysis of the signal found within a thin band (*crown*) of voxels around the edge of the brain, as proposed by [20]. The BOLD time-series were resampled into standard space, generating a *preprocessed BOLD run in MNI152NLin6Asym space*. First, a reference volume and its skull-stripped version were generated using a custom methodology of *fMRIPrep*. The BOLD time-series were resampled onto the following surfaces (FreeSurfer reconstruction nomenclature): *fsnative*, *fsaverage*, *fsaverage5*. All resamplings can be performed with *a single interpolation step* by composing all the pertinent transformations (i.e. head-motion transform matrices, susceptibility distortion correction when available, and co-registrations to anatomical and output spaces). Gridded (volumetric) resamplings were performed using antsApplyTransforms (ANTs), configured with Lanczos interpolation to minimize the smoothing effects of other kernels [21]. Non-gridded (surface) resamplings were performed using mri_vol2surf (FreeSurfer).

**Flanker task**

For each of the Flanker BOLD runs found per subject across sessions, the following preprocessing was performed. First, a reference volume and its skull-stripped version were generated using a custom methodology of *fMRIPrep*. Head-motion parameters with respect to the BOLD reference (transformation matrices, and six corresponding rotation and translation parameters) are estimated before any spatiotemporal filtering using mcflirt (FSL , [13]). BOLD runs were slice-time corrected to 0.976s (0.5 of slice acquisition range 0s-1.95s) using 3dTshift from AFNI [14]. The BOLD time-series (including slice-timing correction when applied) were resampled onto their original, native space by applying the transforms to correct for head-motion. These resampled BOLD time-series will be referred to as *preprocessed BOLD in original space*, or just *preprocessed BOLD*. The BOLD reference was then co-registered to the T1w reference using bbregister (FreeSurfer) which implements boundary-based registration [16]. Co-registration was configured with six degrees of freedom. Several confounding time-series were calculated based on the *preprocessed BOLD*: framewise displacement (FD), DVARS and three region-wise global signals. FD was computed using two formulations following Power (absolute sum of relative motions, [17]) and Jenkinson (relative root mean square displacement between affines, [13]). FD and DVARS are calculated for each functional run, both using their implementations in *Nipype* (following the definitions by [17]). The three global signals are extracted within the CSF, the WM, and the whole-brain masks. Additionally, a set of physiological regressors were extracted to allow for component-based noise correction (*CompCor*, [18]). Principal components are estimated after high-pass filtering the *preprocessed BOLD* time-series (using a discrete cosine filter with 128s cut-off) for the two *CompCor* variants: temporal (tCompCor) and anatomical (aCompCor). tCompCor components are then calculated from the top 2% variable voxels within the brain mask. For aCompCor, three probabilistic masks (CSF, WM and combined CSF+WM) are generated in anatomical space. The implementation differs from that of Behzadi et al. in that instead of eroding the masks by 2 pixels on BOLD space, a mask of pixels that likely contain a volume fraction of GM is subtracted from the aCompCor masks. This mask is obtained by dilating a GM mask extracted from the FreeSurfer’s *aseg* segmentation, and it ensures components are not extracted from voxels containing a minimal fraction of GM. Finally, these masks are resampled into BOLD space and binarized by thresholding at 0.99 (as in the original implementation). Components are also calculated separately within the WM and CSF masks. For each CompCor decomposition, the *k* components with the largest singular values are retained, such that the retained components’ time series are sufficient to explain 50 percent of variance across the nuisance mask (CSF, WM, combined, or temporal). The remaining components are dropped from consideration. The head-motion estimates calculated in the correction step were also placed within the corresponding confounds file. The confound time series derived from head motion estimates and global signals were expanded with the inclusion of temporal derivatives and quadratic terms for each [19]. Frames that exceeded a threshold of 0.5 mm FD or 1.5 standardized DVARS were annotated as motion outliers. Additional nuisance timeseries are calculated by means of principal components analysis of the signal found within a thin band (*crown*) of voxels around the edge of the brain, as proposed by [20]. The BOLD time-series were resampled into standard space, generating a *preprocessed BOLD run in MNI152NLin6Asym space*. First, a reference volume and its skull-stripped version were generated using a custom methodology of *fMRIPrep*. The BOLD time-series were resampled onto the following surfaces (FreeSurfer reconstruction nomenclature): *fsnative*, *fsaverage*, *fsaverage5*. All resamplings can be performed with *a single interpolation step* by composing all the pertinent transformations (i.e. head-motion transform matrices, susceptibility distortion correction when available, and co-registrations to anatomical and output spaces). Gridded (volumetric) resamplings were performed using antsApplyTransforms (ANTs), configured with Lanczos interpolation to minimize the smoothing effects of other kernels [21]. Non-gridded (surface) resamplings were performed using mri_vol2surf (FreeSurfer).

**Emotional Dot-probe task**

For each of the Dot-probe BOLD runs found per subject across sessions, the following preprocessing was performed. First, a reference volume and its skull-stripped version were generated using a custom methodology of *fMRIPrep*. Head-motion parameters with respect to the BOLD reference (transformation matrices, and six corresponding rotation and translation parameters) are estimated before any spatiotemporal filtering using mcflirt (FSL ,[13]). BOLD runs were slice-time corrected to 1.13s (0.5 of slice acquisition range 0s-2.25s) using 3dTshift from AFNI [14]. The BOLD time-series (including slice-timing correction when applied) were resampled onto their original, native space by applying the transforms to correct for head-motion. These resampled BOLD time-series will be referred to as *preprocessed BOLD in original space*, or just *preprocessed BOLD*. The BOLD reference was then co-registered to the T1w reference using bbregister (FreeSurfer) which implements boundary-based registration [16]. Co-registration was configured with six degrees of freedom. Several confounding time-series were calculated based on the *preprocessed BOLD*: framewise displacement (FD), DVARS and three region-wise global signals. FD was computed using two formulations following Power (absolute sum of relative motions, [17]) and Jenkinson (relative root mean square displacement between affines, [13]). FD and DVARS are calculated for each functional run, both using their implementations in *Nipype* (following the definitions by [17]). The three global signals are extracted within the CSF, the WM, and the whole-brain masks. Additionally, a set of physiological regressors were extracted to allow for component-based noise correction (*CompCor*, [18]). Principal components are estimated after high-pass filtering the *preprocessed BOLD* time-series (using a discrete cosine filter with 128s cut-off) for the two *CompCor* variants: temporal (tCompCor) and anatomical (aCompCor). tCompCor components are then calculated from the top 2% variable voxels within the brain mask. For aCompCor, three probabilistic masks (CSF, WM and combined CSF+WM) are generated in anatomical space. The implementation differs from that of Behzadi et al. in that instead of eroding the masks by 2 pixels on BOLD space, a mask of pixels that likely contain a volume fraction of GM is subtracted from the aCompCor masks. This mask is obtained by dilating a GM mask extracted from the FreeSurfer’s *aseg* segmentation, and it ensures components are not extracted from voxels containing a minimal fraction of GM. Finally, these masks are resampled into BOLD space and binarized by thresholding at 0.99 (as in the original implementation). Components are also calculated separately within the WM and CSF masks. For each CompCor decomposition, the *k* components with the largest singular values are retained, such that the retained components’ time series are sufficient to explain 50 percent of variance across the nuisance mask (CSF, WM, combined, or temporal). The remaining components are dropped from consideration. The head-motion estimates calculated in the correction step were also placed within the corresponding confounds file. The confound time series derived from head motion estimates and global signals were expanded with the inclusion of temporal derivatives and quadratic terms for each [19]. Frames that exceeded a threshold of 0.5 mm FD or 1.5 standardized DVARS were annotated as motion outliers. Additional nuisance timeseries are calculated by means of principal components analysis of the signal found within a thin band (*crown*) of voxels around the edge of the brain, as proposed by [20]. The BOLD time-series were resampled into standard space, generating a *preprocessed BOLD run in MNI152NLin6Asym space*. First, a reference volume and its skull-stripped version were generated using a custom methodology of *fMRIPrep*. The BOLD time-series were resampled onto the following surfaces (FreeSurfer reconstruction nomenclature): *fsnative*, *fsaverage*, *fsaverage5*. All resamplings can be performed with *a single interpolation step* by composing all the pertinent transformations (i.e. head-motion transform matrices, susceptibility distortion correction when available, and co-registrations to anatomical and output spaces). Gridded (volumetric) resamplings were performed using antsApplyTransforms (ANTs), configured with Lanczos interpolation to minimize the smoothing effects of other kernels [21]. Non-gridded (surface) resamplings were performed using mri_vol2surf (FreeSurfer).

1. **Further processing of fMRI data**

We used Python version 3.10 and MATLAB version R2023a for further processing of the fMRI data; the code can be found here: https://github.com/NIMH-SDAN/Neural.Efficiency.2025. Time series were extracted from 100 cortical parcels, derived from the Schaefer 17 network atlas [22, 23], and sixteen additional subcortical regions, derived from FreeSurfer segmentation (i.e., bilateral nucleus accumbens, caudate, pallidum, putamen, amygdala, hippocampus, thalamus, and ventral diencephalon). Nuisance regressors parsed out from both rest and task time series included white matter, cerebrospinal fluid, and global signal, and the ICA-AROMA components (non-aggressive version). Task events (Flanker arrow display and face pairs) were additionally regressed out of the task data to minimize spurious associations driven by shared task timing (e.g., inflated estimates between visual and auditory cortices due to simultaneous visual-auditory stimulus presentation)[24]. Functional connectivity was calculated as partial Pearson correlations, which measure direct connectivity between two regions [25], and have been shown to exhibit better test-retest reliability [26] compared to full correlations. Partial correlation coefficients underwent Fisher’s z’-transformation. Preprocessing resulted in one to four pairs (task/rest combination for each time point) of 116x116 connectivity matrices per participant. Neural efficiency per task/rest combination per session was then calculated as a Pearson correlation of the vectorized upper diagonal elements of each pair of task/rest functional connectivity matrices.

1. **Additional analytical information**

All group level models were estimated in RStudio version 2025.09.2. To estimate developmental stability, we used a Bayesian linear mixed-effects approach. We used ICCs with a consistency formulation (ICC[3,1]) with participant as a random effect and Gamma priors [27] and fixed effects for session. This formulation examines consistency in rank rather than absolute value, which accounts for systematic changes over time, such as practice effects.

1. **Additional results**

Correlations between similarity values computed with and without regressing out global signal were high (Flanker: 16-year time point: *r*(41)=.99, *p*<.001, 19-year time point: *r* (41)=.99, *p* <.001; Dot-probe: 16-year time point: *r* (34)=.93,  *p*<.001; 19-year time point: *r* (38)=.98, *p*<.001).

Similarity values for the two task-rest combinations (i.e., Flanker-rest and Dot-probe-rest) correlated at each time point (16-year time point: *r*(31)=.69, *p*<.001; 19-year time point: *r*(32)=.30, *p*=.09).

Using full rather than partial correlations to derive the neural efficiency metric resulted in a lower ICC for the dot Dot-probe task (ICC=.10), but not for the flanker task (ICC=.52).

**References**

1. Tottenham, N., et al., *The NimStim set of facial expressions: Judgments from untrained research participants.* Psychiatry research, 2009. **168**(3): p. 242–249.

2. Esteban, O., et al., *Crowdsourced MRI quality metrics and expert quality annotations for training of humans and machines.* Scientific data, 2019. **6**(1): p. 30.

3. Gorgolewski, K., et al., *Nipype: a flexible, lightweight and extensible neuroimaging data processing framework in python.* Frontiers in neuroinformatics, 2011. **5**: p. 13.

4. Tustison, N.J., et al., *N4ITK: improved N3 bias correction.* IEEE transactions on medical imaging, 2010. **29**(6): p. 1310–1320.

5. Avants, B.B., et al., *Symmetric diffeomorphic image registration with cross-correlation: evaluating automated labeling of elderly and neurodegenerative brain.* Medical image analysis, 2008. **12**(1): p. 26–41.

6. Zhang, Y., M. Brady, and S. Smith, *Segmentation of brain MR images through a hidden Markov random field model and the expectation-maximization algorithm.* IEEE transactions on medical imaging, 2002. **20**(1): p. 45–57.

7. Reuter, M., H.D. Rosas, and B. Fischl, *Highly accurate inverse consistent registration: a robust approach.* Neuroimage, 2010. **53**(4): p. 1181–1196.

8. Dale, A.M., B. Fischl, and M.I. Sereno, *Cortical surface-based analysis: I. Segmentation and surface reconstruction.* Neuroimage, 1999. **9**(2): p. 179–194.

9. Klein, A., et al., *Mindboggling morphometry of human brains.* PLoS computational biology, 2017. **13**(2): p. e1005350.

10. Ciric, R., et al., *TemplateFlow: FAIR-sharing of multi-scale, multi-species brain models.* Nature methods, 2022. **19**(12): p. 1568–1571.

11. Evans, A.C., et al., *Brain templates and atlases.* Neuroimage, 2012. **62**(2): p. 911–922.

12. Fonov, V.S., et al., *Unbiased nonlinear average age-appropriate brain templates from birth to adulthood.* NeuroImage, 2009. **47**: p. S102.

13. Jenkinson, M., et al., *Improved optimization for the robust and accurate linear registration and motion correction of brain images.* Neuroimage, 2002. **17**(2): p. 825–841.

14. Cox, R.W. and J.S. Hyde, *Software tools for analysis and visualization of fMRI data.* NMR in Biomedicine: An International Journal Devoted to the Development and Application of Magnetic Resonance In Vivo, 1997. **10**(4‐5): p. 171–178.

15. Posse, S., et al., *Enhancement of BOLD‐contrast sensitivity by single‐shot multi‐echo functional MR imaging.* Magnetic Resonance in Medicine: An Official Journal of the International Society for Magnetic Resonance in Medicine, 1999. **42**(1): p. 87–97.

16. Greve, D.N. and B. Fischl, *Accurate and robust brain image alignment using boundary-based registration.* Neuroimage, 2009. **48**(1): p. 63–72.

17. Power, J.D., et al., *Methods to detect, characterize, and remove motion artifact in resting state fMRI.* neuroimage, 2014. **84**: p. 320–341.

18. Behzadi, Y., et al., *A component based noise correction method (CompCor) for BOLD and perfusion based fMRI.* Neuroimage, 2007. **37**(1): p. 90–101.

19. Satterthwaite, T.D., et al., *An improved framework for confound regression and filtering for control of motion artifact in the preprocessing of resting-state functional connectivity data.* Neuroimage, 2013. **64**: p. 240–256.

20. Patriat, R., R.C. Reynolds, and R.M. Birn, *An improved model of motion-related signal changes in fMRI.* NeuroImage, 2017. **144**: p. 74–82.

21. Lanczos, C., *Evaluation of noisy data.* Journal of the Society for Industrial and Applied Mathematics, Series B: Numerical Analysis, 1964. **1**(1): p. 76–85.

22. Schaefer, A., et al., *Local-global parcellation of the human cerebral cortex from intrinsic functional connectivity MRI.* Cerebral cortex, 2018. **28**(9): p. 3095–3114.

23. Yeo, B.T., et al., *The organization of the human cerebral cortex estimated by intrinsic functional connectivity.* Journal of neurophysiology, 2011.

24. Cole, M.W., et al., *Task activations produce spurious but systematic inflation of task functional connectivity estimates.* Neuroimage, 2019. **189**: p. 1–18.

25. Bijsterbosch, J., S.M. Smith, and C. Beckmann, *An introduction to resting state fMRI functional connectivity*. 2017: Oxford University Press.

26. Linke, J.O., et al., *Reduced threat-related neural efficiency: a possible biomarker for pediatric anxiety disorders.* American Journal of Psychiatry, 2025: p. appi. ajp. 20241043.

27. Chen, G., et al., *Linear mixed-effects modeling approach to FMRI group analysis.* Neuroimage, 2013. **73**: p. 176–190.
